# Implications for cisgender female underrepresentation, small sample sizes, and misgendering in sport and exercise science research

**DOI:** 10.1371/journal.pone.0291526

**Published:** 2023-11-30

**Authors:** James W. Navalta, Dustin W. Davis, Whitley J. Stone

**Affiliations:** 1 Department of Kinesiology and Nutrition Sciences, University of Nevada, Las Vegas, Las Vegas, Nevada, United States of America; 2 Department of Kinesiology, Recreation, and Sport, Western Kentucky University, Bowling Green, Kentucky, United States of America; Tokat Gaziosmanpasa University Tasliciftlik Campus: Tokat Gaziosmanpasa Universitesi, TURKEY

## Abstract

A sex-data gap, from testing primarily males, results in a lack of scientific knowledge for other groups (females, transgender individuals). It is unknown whether typical recruitment and participant characterization causes incorrect statistical decisions, and three factors were evaluated: 1) underrepresenting cisgender females, 2) recruiting small sample sizes, 3) misgendering. Data from the National Health and Nutrition Examination Survey (2003–2004) were evaluated for sex differences after removing missing values (*N* = 3,645; F = 1,763). Disparities were determined by utilizing sample sizes common in sport and exercise science research; mean sample size *N* = 187, median sample size *N* = 20. Participants were randomly allocated into datasets in an imbalanced manner (33.5% females, 66.5% males). Potential effects of misgendering were determined at rates of 2% and 5%. Differences between the complete data set and expected decisions were conducted through Chi-squared (χ^2^) goodness of fit with significance at *p* < .05. When the entire dataset was evaluated as if a sex testing disparity was present, decisions were not altered (χ^2^ = .52, *p* = .47). Differences were observed for mean sample size (χ^2^ = 4.89, *p* = .027), median sample size (χ^2^ = 13.52, *p* < .001), and misgendering at 2% (χ^2^ = 13.52, *p* = < .001) and 5% (χ^2^ = 13.52, *p* = < .001). Recruitment practices in sport and exercise science research should be revisited, as testing primarily cisgender males has consequences, particularly in small sample sizes. Misgendering participants also has consequences on ultimate decisions and interpretations of data, regardless of sample size. Inclusiveness is needed in helping all individuals feel valued and respected when participating in sport and exercise science research.

## Introduction

Deficiencies in scientific knowledge that occur due to unequal recruiting and testing practices between males and females (typically cisgender in the literature, although not explicitly stated) are known as the “sex-data gap” [1, 2]. Issues highlighting the sex-data gap in sport and exercise science were identified by Costello et al., who reported a disparity (39% of participants were females, 61% of participants were males) in three leading journals [3]. A follow-up by Cowley et al. doubled the number of journals and found that little changed in testing disparities during the ensuing six-year period (34% of participants were females, 66% of participants were males) [1]. The most recent investigation into the sex-data gap by Garver et al. reported similar tendencies in a student-focused journal (36% of participants were females, 64% of participants were males) [2]. The consequences of continued underrepresentation are impactful, as females experience sport and exercise and associated recovery differently than males [1] and lack appropriate scientific data to make informed decisions about adjusting important training-based variables [3]. There is a clear need to be inclusive when carrying out high-quality investigations [2].

The literature contains ample evidence of sex differences between females and males, particularly when body composition measures are evaluated [4]. For example, in a survey of healthy Han adults from Shaanxi Province, China, significant differences were noted for body mass (females = 58 ± 9 kilograms [kg], males = 74 ± 11 kg, *p* < .001) but not body mass index (BMI) (females = 23 ± 3 kg•meter^−2^ (kg•m^−2^), males = 25 ± 3 kg•m^−2^, *p* = .706) [5]. Significant sex differences for BMI, however, have been reported between middle-aged African Americans (females = 31 ± 5 kg•m^−2^, males = 29 ± 5 kg•m^−2^, *p* < .05) [6]. Differences in subscapular and triceps skinfold measures were noted in healthy young Japanese adults (subscapular: females = 19 ± 5 millimeters [mm], males = 12 ± 4 mm; triceps: females = 20 ± 5 mm, males = 10 ± 5 mm) [7]. Waist girth has been shown to be significantly lower in females from New Zealand in the early- (70 ± 9 centimeters [cm]), late- (74 ± 9 cm), and post-pubertal stage (79 ± 14 cm) than males (early- = 72 ± 10 cm, late- = 79 ± 8 cm, post-pubertal = 84 ± 8 cm, all comparisons *p* < .001) [8]. Thus, based on the available literature, there is strong evidence of body composition differences between females and males. It is unknown whether typical sport and exercise science recruiting and sample size testing may ultimately affect outcomes and decisions. Considering the sex-data gap and frequency of binary participant characterization (cisgender female, cisgender male), the field of sport and exercise science may be facing an invisible issue. It is unknown whether the typical approaches to recruitment and participant characterization have caused incorrect statistical decisions about differences in body composition among genders. Three factors may be at play: underrepresenting cisgender females, recruiting small sample sizes, and misgendering.

While sample sizes can influence significance testing and subsequent interpretation, such decisions require understanding and nuance [9]. Marsh et al. investigated 30 well-known models of confirmatory factor analysis from the Self Description Questionnaire and reported that sample size substantially affected all but one index [10]. Adequate sample size is an issue that has plagued sport and exercise science research for the past half century [11]. The issue persists, as an evaluation of 120 randomly selected manuscripts published in the *Journal of Sports Sciences* found a median sample size of 19 [12], and a 30-year evaluation of 676 investigations published in the *Journal of Applied Biomechanics* reported the majority (71%) utilized 2–20 participants [13]. A recent investigation of 806 studies on human subjects published in the *International Journal of Exercise Science* (IJES) over a 14-year period had a median sample size of 20 participants [2]. Other fields are concerned with the sampling of rare species, which naturally have a small number of occurrences [14]. However, it cannot be argued that female participants in sport and exercise science have a similar distribution. To our knowledge, the intersection of recruiting traditionally small sample sizes with a disparity of female participants has not been evaluated in sport and exercise science research.

Another area of concern in sport and exercise science research is appropriately representing individuals who do not identify into a gender-normative binary definition of sex. Garver et al. reported that, of 151,043 participants evaluated in the IJES self-study, one participant identified as transgender, three identified as other, and one declined to identify their gender [2]. The current estimation is that as much as 2% [15] to 5% [16] of the U.S. population identifies as transgender or nonbinary. Thus, between 3,031 and 7,552 participants in the IJES self-study could have been classified as the incorrect gender, or as a gender other than cisgender female or cisgender male. Based on the available literature, it seems reasonable to conclude that many sport and exercise science researchers do not have a mechanism established to evaluate the gender constitution of their recruited samples by a method other than partitioning into female and male (implying cisgender female and cisgender male). It also seems reasonable to conclude that many participants included in sport and exercise science research may be misgendered. When a person is described in a language that does not coincide with their gender identity, misgendering occurs [17]. In healthcare, the consequences of misgendering include individuals not utilizing healthcare [18], delays in seeking and obtaining medical care leading to increased emergency treatment [19], and receiving incompetent care or being denied treatment [20]. To our knowledge, the consequences of misgendering in sport and exercise science research has not been evaluated.

Several questions remain to be addressed in the sport and exercise science literature: 1) the effect of the consistent recruiting imbalance between females and males, 2) the impact of small sample sizes, and 3) the consequences of misgendering. The purpose of this investigation was to determine how statistical decisions may change when considering the effect of these three questions. Body composition measures were utilized because studies have reported significant differences between people characterized according to the gender-normative binary definition of sex. It was hypothesized that recruiting an imbalanced ratio of females to males, utilizing small sample sizes, and misgendering individuals would all negatively affect decisions regarding between-sex differences in body composition measures.

## Methods

### Participants

Fully anonymized data for this study were obtained from public use datasets of the National Health and Nutrition Examination Survey (NHANES) released from 2003 to 2004 (National Center for Health Statistics Research Ethics Review Board approval protocol #98–12). During the written informed consent process, survey participants were guaranteed that collected data would be used only for the stated purpose and would not be disclosed or released in accordance with section 308(d) of the Public Health Service Act (42 U.S.C. 242m). Conducted by the Centers for Disease Control, the NHANES survey collects representative information from the United States population in the form of both survey and physical examination measures relating to health and nutritional status. Details of the survey and laboratory procedures are available elsewhere [21, 22].

The initial dataset contained information from 9,041 participants. After participants aged 0–17 were removed, 4,965 participants aged 18–85 years (y) old remained (*n* = 2,587 females, *n* = 2,378 males). Body composition data extracted for the purpose of this analysis included the dependent variables of BMI, subscapular skinfold, triceps skinfold, waist girth, and body mass. Cases were removed (*n* = 496 males, *n* = 824 females) if any dependent variable was missing, resulting a final dataset of 3,645 participants (see Table 1).

**Table 1:** Demographic information of participants included in the final dataset.

	Age (y)	BMI (kg•m^−2^)	Subscapular skinfold (mm)	Triceps skinfold (mm)	Waist girth (cm)	Body Mass (kg)
Female (*n* = 1,763)	46.1 (21.6)	26.0 (4.9)	20.0 (7.7)	23.0 (7.3)	90.2 (12.7)	67.6 (13.5)
Male (*n* = 1,882)	46.7 (21.2)	26.4 (4.4)	18.6 (7.2)	13.7 (6.1)	95.6 (13.1)	80.8 (15.2)

Values are means (standard deviations). Y: years; kg•m^−2^: kilograms per meter squared; mm: millimeters; cm: centimeters

### Protocol

Our initial analysis was to test for sex differences for each dependent variable in the overall sample, as well as by age category (18–29 y, 30–39 y, 40–49 y, 50–59 y, 60–69 y, 70–85 y). This overall sample served as the baseline for differences (*p* < 0.05) and effect size (small, medium, large) decisions.

We then tested to determine whether an imbalance in recruiting fewer females (33.5% females versus 66.5% males), as has been consistently shown in the sport and exercise science literature [1–3], would affect decisions that were made at baseline. To do this, the entire male sample was retained, and two-thirds of the female sample was randomly removed using the random function in Microsoft Excel (Version 16.72, Microsoft Corporation, Redmond, WA). The analysis was conducted overall as well as by age category.

Next, the disparity in recruiting and testing was determined by utilizing sample sizes commonly employed in the sport and exercise science literature. According to Garver et al. [2] the mean sample size was 187 participants (referred to as “Large Ratio” moving forward) and the median sample size was 20 participants (subsequently referred to as “Small Ratio”). Participants were randomly allocated into the Large Ratio and Small Ratio datasets in an imbalanced manner (33.5% females, 66.5% males) using the random function in Microsoft Excel. The analyses for Large Ratio and Small Ratio were conducted overall as well as by age category.

Finally, the potential effects of misgendering in the sample were determined. These effects were determined at a rate of 2% [15], and at a rate of 5% [16] in both Large Ratio and Small Ratio. As was done for the random allocation disparity described above, misgendered participants were randomly determined. For example, transgender females who were classified as males, were allocated into the dataset as females. Similarly, transgender males who were classified as females, were allocated into the dataset as males. The analyses for Large Ratio with misgendering occurring at 2% and 5% and Small Ratio at 5% were conducted overall as well as by age category. Because sport and exercise science investigations recruit sexes in an unequal ratio, the number of transgender people randomly included in these datasets followed the same distribution (i.e., 66.5% were added as transgender females, and 33.5% were added as transgender males). Because the Small Ratio with misgendering occurring at 2% represented less than a whole number of misgendered participants, the consequences of misgendering a single participant were determined, both in the case of a misgendered male and in the case of a misgendered female. These analyses were conducted overall as well as by age category.

### Statistical analysis

Differences in the binary sex comparison between females and males was conducted through a one-sided independent *t*-test for each dependent variable (IBM SPSS Statistics, Version 28.0.1.0, IBM Corp., Armonk, NY). Significance was accepted at the *p* ≤ 0.05 level. Effect sizes were determined through Cohen’s *d*, with small = 0.00–0.49, medium = 0.50–0.79, and large ≥ 0.80 [23].

Differences between the complete data set and expected decisions for subsequent iterations were conducted through Chi-squared (χ^2^) goodness of fit analysis in Microsoft Excel. It was expected that less than 5% of the decisions would be affected (significant or not significant at the *p* < 0.05 level, and effect size classification changes as small, medium, or large). Effect size Phi (φ) interpretation was trivial < 0.1, small = 0.1–0.29, medium = 0.3–0.49, and large ≥ 0.5 [23].

## Results

### Baseline

The initial comparisons for body composition measures between all females and males included from the NHANES data are shown in Table 2. Significant differences were observed for all measures in the overall group. When age groups for this dataset were considered, significant differences were noted for all variables for 18–29 y. No significant difference in BMI was observed for 30–39 y, 40–49 y, 50–59 y, 60–69 y, or 70+ y. Differences between sexes existed for all other dependent variables by age group except for subscapular skinfold in 60–69 y.

**Table 2:** Baseline comparisons between females and males for NHANES body composition measures using the entire identified dataset.

	Sample Size	BMI (kg•m^−2^)	Subscapular Skinfold (mm)	Triceps Skinfold (mm)	Waist Girth (cm)	Body Mass (kg)
Female	1,763	26.0 (4.9)	20.0 (7.7)	23.0 (7.3)	90.2 (12.7)	67.6 (13.5)
Male	1,882	26.4 (4.3)	18.6 (7.2)	13.7 (6.1)	95.6 (13.1)	80.8 (15.2)
*p*-value		**0.012**	**< 0.001**	**< 0.001**	**< 0.001**	**< 0.001**
Cohen’s *d*		0.075 (small)	0.186 (small)	1.387 (large)	0.423 (small)	0.919 (large)
18–29 Year						
Female	538	24.5 (4.6)	18.9 (7.9)	21.2 (7.3)	85.1 (12.9)	64.7 (12.8)
Male	536	25.0 (4.7)	16.0 (7.5)	13.2 (7.0)	87.6 (13.0)	78.6 (16.3)
*p*-value		**0.039**	**< 0.001**	**< 0.001**	**< 0.001**	**< 0.001**
Cohen’s *d*		0.108 (small)	0.373 (small)	1.121(large)	0.194 (small)	0.945 (large)
30–39 Year						
Female	267	25.9 (5.0)	20.8 (7.5)	23.5 (7.2)	89.5 (12.9)	69.1 (14.2)
Male	265	26.6 (4.0)	18.8 (6.6)	13.4 (5.4)	93.6 (11.0)	81.4 (14.4)
*p*-value		0.055	**< 0.001**	**< 0.001**	**< 0.001**	**< 0.001**
Cohen’s *d*		0.138 (small)	0.282 (small)	1.596 (large)	0.345 (small)	0.861 (large)
40–49 Year						
Female	213	26.8 (5.5)	21.9 (8.0)	24.5 (7.5)	90.3 (12.2)	70.4 (15.1)
Male	269	27.1 (3.9)	20.2 (7.0)	13.8 (5.6)	97.1 (10.7)	83.8 (13.6)
*p*-value		0.224	**0.008**	**< 0.001**	**< 0.001**	**< 0.001**
Cohen’s *d*		0.070 (small)	0.223 (small)	1.637 (large)	0.600 (medium)	0.932 (large)
50–59 Year						
Female	161	26.4 (4.8)	22.1 (7.6)	24.8 (7.0)	90.7 (12.2)	69.2 (12.6)
Male	196	26.9 (4.1)	20.1 (6.8)	13.6 (5.7)	99.5 (11.2)	83.7 (14.4)
*p*-value		0.123	**0.005**	**< 0.001**	**< 0.001**	**< 0.001**
Cohen’s *d*		0.124 (small)	0.280 (small)	1.778 (large)	0.750 (medium)	1.063 (large)
60–69 Year						
Female	251	27.9 (5.1)	21.8 (7.2)	25.2 (6.9)	95.7 (11.8)	72.0 (13.7)
Male	231	27.7 (4.6)	21.3 (6.8)	14.5 (6.1)	102.2 (12.2)	84.2 (15.7)
*p*-value		0.378	0.201	**< 0.001**	**< 0.001**	**< 0.001**
Cohen’s *d*		0.028 (small)	0.076 (small)	1.631 (large)	0.547 (medium)	0.832 (large)
70+ Year						
Female	332	26.5 (4.2)	17.7 (6.9)	22.0 (6.7)	94.3 (10.3)	65.3 (11.4)
Male	385	26.6 (4.0)	18.7 (6.5)	14.1 (5.7)	101.22 (11.3)	78.1 (14.2)
*p*-value		0.485	**0.019**	**< 0.001**	**< 0.001**	**< 0.001**
Cohen’s *d*		0.003 (small)	0.157 (small)	1.269 (large)	0.632 (medium)	0.984 (large)

Values are means (standard deviations). Cohen’s *d* interpreted as small = 0.00–0.49, medium = 0.50–0.79, and large ≥ 0.80 [23]. NHANES: National Health and Nutrition Examination Survey; BMI: body mass index in kilograms (kg)•meter^−2^; mm: millimeters; cm: centimeters.

### Effect of sex disparity

When the entire dataset was evaluated as if a testing disparity was present for sex, decisions were not altered for the overall group. While changes in decisions were noted for two age groups (BMI in 18–29 y and 40–49 y) the impact was not significant (see Table 3 and S1A Table).

**Table 3:** Summary table representing changes (in gray) compared to baseline for the entire dataset (*N* = 2830), large dataset (*N* = 187), and small dataset (*N* = 20) as if a testing disparity was present for sex.

		BMI (kg•m^−2^)	Subscapular Skinfold (mm)	Triceps Skinfold (mm)	Waist Girth (cm)	Body Mass (kg)
Entire dataset with sex disparity (S1 Table A)	Overall	**0.05**	**< 0.001**	**< 0.001**	**< 0.001**	**< 0.001**
	18–29 Year	0.064	**< 0.001**	**< 0.001**	**< 0.001**	**< 0.001**
	30–39 Year	**0.045**	**0.002**	**< 0.001**	**< 0.001**	**< 0.001**
	40–49 Year	0.344	**0.008**	**< 0.001**	**< 0.001**	**< 0.001**
	50–59 Year	0.069	**0.012**	**< 0.001**	**< 0.001**	**< 0.001**
	60–69 Year	0.189	0.417	**< 0.001**	**< 0.001**	**< 0.001**
	70+ Year	0.447	**0.031**	**< 0.001**	**< 0.001**	**< 0.001**
Large dataset with sex disparity (S1 Table B)	Overall	0.358	**0.038**	**< 0.001**	**0.003**	**< 0.001** 0.774 (medium)
	18–29 Year	0.087	0.065	**< 0.001**	**0.017**	**< 0.001**
	30–39 Year	0.098	**0.029**	**< 0.001**	**0.019**	**< 0.001**
	40–49 Year	0.086	0.327	**< 0.001**	**< 0.001**	**< 0.001**
	50–59 Year	0.354	**0.007**	**< 0.001**	**< 0.001**	**< 0.001**
	60–69 Year	0.343	0.067	**< 0.001**	**< 0.001**	**< 0.001** 0.785 (medium)
	70+ Year	0.283	0.341	**< 0.001**	**0.003** 0.429 (small)	**< 0.001**
Small dataset with sex disparity (S1 Table C)	Overall	0.438	0.242	**0.04**	0.329	**0.038**
	18–29 Year	**0.017** 0.910 (large)	0.331	0.236 0.334 (small)	**0.03** 0.821 (large)	**< 0.001**
	30–39 Year	**0.045** 0.851 (large)	0.152 0.529 (medium)	**0.005**	**0.011** 1.113 (large)	**< 0.001**
	40–49 Year	0.116	**0.002** 0.710 (medium)	0.367	0.367 0.159 (small)	0.178 0.50 (medium)
	50–59 Year	0.142	0.403	**0.001**	**0.005** 1.441 (large)	**0.001**
	60–69 Year	0.168	0.364	**0.004**	**0.008** 1.542 (large)	**< 0.001**
	70+ Year	0.31	0.482	**0.011**	0.376 0.142 (small)	0.053

BMI: body mass index in kilograms (kg)•meter^−2^; mm: millimeters; cm: centimeters.

When a large dataset (*N* = 187) was evaluated as if a testing disparity was present for sex, a significant difference compared to baseline was observed for the overall randomly drawn sample (see S1B Table). While decision changes were noted for one variable in specific age groups (subscapular skinfold: 18–29 y, 40–49 y; BMI: 30–39 y; body mass: 60–69 y; waist girth: 70+ y) the impact was not significant.

When a small dataset (*N* = 20) was evaluated as if a testing disparity was present for sex, a significant difference compared to baseline was observed for the overall randomly drawn sample (see S1C Table). Significant differences in decisions and interpretation were also noted for every age category with the exception of the 60–69 y group.

### Effect of misgendering

When a large dataset (*N* = 187) was evaluated as if 2% of the sample were misgendered, the results are shown in Table 4. It is important to note that differences were not compared to baseline, but to the large dataset that included sex disparities (i.e., S1B Table). A significant difference was observed for the overall randomly drawn sample. While decision changes were noted for at least one variable (generally waist girth) in all age groups but one (40–49 y), it was only significant in the 50–59 y group.

**Table 4:** Female and male NHANES body composition measures using a large dataset (*N* = 187) as if 2% of the sample were misgendered. Expected results compared to sex disparities in a large dataset were conducted using χ^2^.

	Sample Size	BMI (kg•m^2^)	Subscapular Skinfold (mm)	Triceps Skinfold (mm)	Waist Girth (cm)	Body Mass (kg)
Female	62	27.0 (4.6)	20.4 (6.9)	23.5 (7.3)	92.2 (11.5)	72.1 (13.6)
Male	125	26.4 (3.9)	18.7 (7.0)	13.6 (6.0)	95.2 (11.9)	80.6 (13.5)
*p*-value		0.19	0.052	**< 0.001**	0.052	**< 0.001**
Cohen’s *d*		0.145 (small)	0.254 (small)	1.544 (large)	0.521 (medium)	0.629 (medium)
χ^2^ Result	χ^2^ (1, *N* = 10) = 13.52, *p* = <0.001, φ = 1.16 (large)					
18–29 Year						
Female	62	24.7 (4.4)	18.5 (7.9)	20.1 (8.6)	85.3 (11.1)	64.7 (12.7)
Male	125	25.0 (4.7)	16.5 (7.9)	13.8 (7.4)	87.9 (13.1)	78.9 (16.8)
*p*-value		0.334	0.053	**< 0.001**	0.078	**< 0.001**
Cohen’s *d*		0.065 (small)	0.254 (small)	0.856 (large)	0.209 (small)	0.909 (large)
χ^2^ Result	χ^2^ (1, *N* = 10) = 0.56, *p* = 0.455, φ = 0.236 (small)					
30–39 Year						
Female	62	25.6 (4.3)	20.5 (7.5)	23.6 (7.4)	87.9 (9.9)	68.3 (12.2)
Male	125	26.5 (4.1)	18.5 (6.6)	13.7 (5.4)	93.8 (10.9)	81.8 (13.5)
*p*-value		0.084	**0.04**	**< 0.001**	**< 0.001**	**< 0.001**
Cohen’s *d*		0.219 (small)	0.288(small)	1.612 (large)	0.559 (medium)	1.031 (large)
χ^2^ Result	χ^2^ (1, *N* = 10) = 0.56, *p* = 0.455, φ = 0.236 (small)					
40–49 Year						
Female	62	26.7 (4.6)	20.7 (7.7)	23.8 (8.2)	89.8 (10.7)	70.0 (13.0)
Male	125	27.2 (3.9)	20.0 (6.8)	13.6 (52)	97.3 (10.5)	84.6 (13.9)
*p*-value		0.234	0.278	**< 0.001**	**< 0.001**	**< 0.001**
Cohen’s *d*		0.120 (small)	0.096 (small)	1.600 (large)	0.713 (medium)	1.070 (large)
χ^2^ Result	χ^2^ (1, *N* = 10) = 0.52, *p* = 0.473, φ = 0.227 (small)					
50–59 Year						
Female	62	26.4 (4.7)	21.3 (7.6)	23.7 (7.5)	91.2 (12.6)	70.0 (13.6)
Male	125	27.4 (3.9)	21.2 (6.8)	13.8 (5.8)	101.0 (11.1)	85.6 (14.2)
*p*-value		0.066	0.466	**< 0.001**	**< 0.001**	**< 0.001**
Cohen’s *d*		0.250 (small)	0.291 (small)	1.553 (large)	0.847 (large)	1.115 (large)
χ^2^ Result	χ^2^ (1, *N* = 10) = 4.89**, *p* = 0.027**, φ = 0.700 (large)					
60–69 Year						
Female	62	27.4 (4.4)	20.7 (7.1)	24.4 (6.9)	94.7 (11.7)	72.1 (13.5)
Male	125	27.8 (4.5)	20.8 (6.9)	14.3 (5.9)	102.0 (12.1)	84.3 (15.8)
*p*-value		0.281	0.449	**< 0.001**	**< 0.001**	**< 0.001**
Cohen’s *d*		0.090 (small)	0.020 (small)	1.606 (large)	0.606 (medium)	0.814 (large)
χ^2^ Result	χ^2^ (1, *N* = 10) = 0.56, *p* = 0.455, φ = 0.236 (small)					
70+ Year						
Female	62	26.9 (4.6)	17.9 (6.0)	21.2 (7.7)	95.1 (10.9)	66.3 (13.0)
Male	125	26.4 (3.9)	18.5 (6.4)	13.7 (4.5)	100.6 (11.0)	77.5 (14.5)
*p*-value		0.213	0.294	**< 0.001**	**0.001**	**< 0.001**
Cohen’s *d*		0.181 (small)	0.083 (small)	1.3001 (large)	0.505 (medium)	0.803 (large)
χ^2^ Result	χ^2^ (1, *N* = 10) = 0.56, *p* = 0.455, φ = 0.236 (small)					

Values are means (standard deviations). Cohen’s *d* interpreted as small = 0.00–0.49, medium = 0.50–0.79, and large ≥ 0.80 [23]. Effect size φ interpreted as trivial <0.1, small = 0.1–0.29, medium = 0.30–49, and large ≥ 0.5 [23]. Gray indicates a different decision or interpretation than initially made at baseline. NHANES: National Health and Nutrition Examination Survey; χ^2^: chi-squared; BMI: body mass index in kilograms(kg)•meter^−2^; mm: millimeters; cm: centimeters.

When a large dataset (*N* = 187) was evaluated as if 5% of the sample were misgendered, the results are shown in Table 5. Differences were not compared to baseline, but to the large dataset that included sex disparities (i.e., S1B Table). A significant difference was observed for the overall randomly drawn sample. Significant differences were also noted for each age group except 50–59 y, and 60–69 y. Variables most commonly affected were waist girth and body mass.

**Table 5:** Female and male NHANES body composition measures using a large dataset (*N* = 187) as if 5% of the sample were misgendered. Expected results compared to sex disparities in a large dataset were conducted using χ^2^.

	Sample Size	BMI (kg•m^−2^)	Subscapular Skinfold (mm)	Triceps Skinfold (mm)	Waist Girth (cm)	Body Mass (kg)
Female	62	27.4 (4.1)	21.8 (7.6)	23.8 (7.0)	94.6 (12.2)	74.0 (12.3)
Male	125	26.4 (4.6)	18.2 (6.2)	13.8 (5.9)	95.9 (12.7)	80.4 (15.3)
*p*-value		0.066	**< 0.001**	**< 0.001**	0.249	**0.001**
Cohen’s *d*		0.079 (small)	0.531 (medium)	1.600 (large)	0.104 (small)	0.448 (small)
χ^2^ Result	χ^2^ (1, *N* = 10) = 13.520, ***p* < 0.001**, φ = 1.16 (large)					
18–29 Year						
Female	62	24.8 (4.3)	18.6 (7.3)	21.4 (7.5)	86.3 (12.9)	67.6 (14.3)
Male	125	24.6 (4.6)	15.7 (7.1)	13.2 (6.7)	86.7 (12.4)	76.9 (17.8)
*p*-value		0.357	**0.006**	**< 0.001**	0.426	**< 0.001**
Cohen’s *d*		0.048 (small)	0.404 (small)	1.180 (large)	0.030 (small)	0.573 (medium)
χ^2^ Result	χ^2^ (1, *N* = 10) = 13.520, ***p* < 0.001**, φ = 1.16 (large)					
30–39 Year						
Female	62	27.0 (5.6)	20.8 (7.9)	24.0 (8.7)	92.1 (14.1)	73.5 (15.5)
Male	125	26.5 (4.1)	18.7 (6.3)	13.4 (5.0)	93.8 (11.0)	80.1 (14.3)
*p*-value		0.258	0.3	**< 0.001**	0.201	**0.002**
Cohen’s *d*		0.112 (small)	0.310 (small)	1.648 (large)	0.142 (small)	0.448 (small)
χ^2^ Result	χ^2^ (1, *N* = 10) = 4.89, ***p* = 0.027**, φ = 0.700 (large)					
40–49 Year						
Female	62	27.3 (5.3)	21.8 (8.1)	24.0 (8.3)	91.9 (13.2)	73.8 (16.4
Male	125	27.1 (3.9)	19.8 (7.0)	13.9 (6.1)	96.3 (11.3)	80.0 (14.7)
*p*-value		0.401	0.05	**< 0.001**	**0.013**	**< 0.001**
Cohen’s *d*		0.039 (small)	0.270 (small)	1.454 (large)	0.371 (small)	0.670 (medium)
χ^2^ Result	χ^2^ (1, *N* = 10) = 13.520, ***p* < 0.001**, φ = 1.16 (large)					
50–59 Year						
Female	62	27.3 (5.2)	23.0 (8.2)	24.2 (7.5)	92.3 (13.8)	73.2 (15.2)
Male	125	26.7 (4.1)	20.0 (6.4)	13.8 (5.8)	98.7 (10.5)	82.6 (14.6)
*p*-value		0.212	**0.004**	**< 0.001**	**< 0.001**	**< 0.001**
Cohen’s *d*		0.124 (small)	0.418 (small)	1.617 (large)	0.546 (medium)	0.634 (medium)
χ^2^ Result	χ^2^ (1, *N* = 10) = 0.558, *p* = 0.455, φ = 0.236 (small)					
60–69 Year						
Female	62	27.7 (5.3)	22.4 (7.1)	24.5 (6.6)	95.5 (10.8)	73.3 (15.0)
Male	125	27.8 (4.5)	20.7 (6.5)	14.6 (6.6)	102.0 (11.7)	83.8 (14.9)
*p*-value		0.445	0.065	**< 0.001**	**< 0.001**	**< 0.001**
Cohen’s *d*		0.023 (small)	0.244 (small)	1.505 (large)	0.571 (medium)	0.702 (medium)
χ^2^ Result	χ^2^ (1, *N* = 10) = 0.515, *p* = 0.473, φ = 0.227 (small)					
70+ Year						
Female	62	27.2 (3.7)	19.1 (6.2)	22.5 (6.2)	95.7 (10.3)	68.5 (12.0)
Male	125	26.6 (4.3)	18.9 (6.7)	14.1 (6.4)	101.5 (11.6)	77.8 (15.4)
*p*-value		0.182	0.415	**< 0.001**	**< 0.001**	**< 0.001**
Cohen’s *d*		0.134 (small)	0.033 (small)	1.334 (large)	0.525 (medium)	0.648 (medium)
χ^2^ Result	χ^2^ (1, *N* = 10) = 4.89, ***p* = 0.027**, φ = 0.700 (large)					

Values are means (standard deviations). Cohen’s *d* interpreted as small = 0.00–0.49, medium = 0.50–0.79, and large ≥ 0.80 [23]. Effect size φ interpreted as trivial <0.1, small = 0.1–0.29, medium = 0.30–0.49, and large ≥ 0.5 [23]. Gray indicates a different decision or interpretation than initially made at baseline. NHANES: National Health and Nutrition Examination Survey; χ^2^: chi-squared; BMI: body mass index in kilograms(kg)•meter^−2^; mm: millimeters; cm: centimeters.

When a small dataset (*N* = 20) was evaluated as if a single female was misgendered, the results are shown in Table 6. It is noted that differences were not compared to baseline, but to the small dataset that included sex disparities (i.e., S1C Table). No significant difference was observed for the overall randomly drawn sample. Significant differences were observed for four age groups (18–29 y, 30–39 y, 60–69 y, and 70+ y).

**Table 6:** Female and male NHANES body composition measures using a small dataset (*N* = 20) as if a single female participant was misgendered. Expected results compared to sex disparities in a small dataset were conducted using χ^2^.

	Sample Size	BMI (kg•m^−2^)	Subscapular Skinfold (mm)	Triceps Skinfold (mm)	Waist Girth (cm)	Body Mass (kg)
Female	7	26.3 (5.4)	16.9 (6.2)	19.4 (6.6)	91.9 (13.3)	68.7 (9.5)
Male	13	26.78 (4.8)	19.5 (8.4)	12.4 (4.8)	98.4 (15.0)	80.7 (14.4)
*p*-value		0.43	0.226	**0.033**	0.337	**0.039**
Cohen’s *d*		0.088 (small)	0.329 (small)	1.285 (large)	0.449 (small)	0.925 (large)
χ^2^ Result	χ^2^ (1, *N* = 10) = 0.515, *p* = 0.473, φ = 0.227 (small)					
18–29 Year						
Female	7	25.9 (3.6)	17.1 (8.6)	18.2 (8.7)	86.6 (6.5)	70.2 (11.5)
Male	13	24.6 (3.9)	13.5 (5.6)	11.1 (6.4)	85.5 (12.7)	78.5 (18.3)
*p*-value		0.236	0.175	**0.044**	0.402	0.116
Cohen’s *d*		0.338 (small)	0.527 (medium)	0.979 (large)	0.098 (small)	0.505 (medium)
χ^2^ Result	χ^2^ (1, *N* = 10) = 155.4, ***p* < 0.001**, φ = 3.942 (large)					
30–39 Year						
Female	7	23.5 (4.5)	16.1 (9.3)	17.1 (7.8)	82.5 (10.8)	66.8 (14.9)
Male	13	26.8 (5.3)	18.1 (6.6)	14.5 (7.5)	92.4 (11.9)	81.4 (15.6)
*p*-value		0.083	0.318	0.241	**0.04**	**0.03**
Cohen’s *d*		0.647 (medium)	0.255 (small)	0.345 (small)	0.859 (large)	0.947 (large)
χ^2^ Result	χ^2^ (1, *N* = 10) = 43.65, ***p* < 0.001**, φ = 2.100 (large)					
40–49 Year						
Female	7	29.0 (2.2)	24.7 (7.5)	24.1 (6.3)	96.6 (10.1)	74.2 (10.3)
Male	13	27.3 (4.8)	20.3 (7.3)	12.8 (4.5)	98.0 (12.5)	83.4 (15.1)
*p*-value		0.149	0.114	**0.001**	0.391	0.063
Cohen’s *d*		0.408 (small)	0.601 (medium)	2.195 (large)	0.123 (small)	0.671 (medium)
χ^2^ Result	χ^2^ (1, *N* = 10) = 0.515, *p* = 0.473, φ = 0.227 (small)					
50–59 Year						
Female	7	26.0 (4.8)	22.2 (6.8)	23.8 (9.4)	90.1 (11.9)	68.6 (11.9)
Male	13	27.9 (2.8)	21.5 (4.8)	13.2 (5.7)	102.7 (8.5)	88.4 (10.9)
*p*-value		0.182	0.408	**0.012**	**0.017**	**0.002**
Cohen’s *d*		0.528 (medium)	0.125 (small)	1.484 (large)	1.297 (large)	1.762 (large)
χ^2^ Result	χ^2^ (1, *N* = 10) = 0.558, *p* = 0.455, φ = 0.236 (small)					
60–69 Year						
Female	7	28.7 (6.1)	20.6 (6.1)	21.5 (4.9)	100.2 (17.4)	75.6 (19.6)
Male	13	26.5 (4.6)	20.7 (8.3)	15.0 (8.3)	97.2 (12.7)	79.2 (16.1)
*p*-value		0.221	0.498	**0.02**	0.349	0.343
Cohen’s *d*		0.412 (small)	0.002 (small)	0.889 (large)	0.207 (small)	0.208 (small)
χ^2^ Result	χ^2^ (1, *N* = 10) = 26.44, ***p* < 0.001**, φ = 1.626 (large)					
70+ Year						
Female	7	28.2 (5.0)	18.3 (6.0)	21.5 (6.5)	96.7 (10.2)	69.2 (12.4)
Male	13	26.1 (4.0)	19.7 (8.5)	13.5 (4.9)	101.5 (9.0)	77.5 (12.1)
*p*-value		0.182	0.332	**0.009**	0.161	0.088
Cohen’s *d*		0.479 (small)	0.187 (small)	1.466 (large)	0.506 (medium)	0.679 (medium)
χ^2^ Result	χ^2^ (1, *N* = 10) = 4.89, ***p* = 0.027**, φ = 0.700 (large)					

Values are means (standard deviations). Cohen’s *d* interpreted as small = 0.00–0.49, medium = 0.50–0.79, and large ≥ 0.80 [23]. Effect size j interpreted as trivial <0.1, small = 0.1–0.29, medium = 0.30–0.49, and large ≥ 0.5 [23]. Gray indicates a different decision or interpretation than initially made at baseline. NHANES: National Health and Nutrition Examination Survey; χ^2^: chi-squared; BMI: body mass index in kilograms(kg)•meter^−2^; mm: millimeters; cm: centimeters.

When a small dataset (*N* = 20) was evaluated as if a single male was misgendered, the results are shown in Table 7. Differences were not compared to baseline, but to the small dataset that included sex disparities (i.e., S1C Table). A significant difference was observed for the overall randomly drawn sample. Significant differences were observed for four age groups (18–29 y, 30–39 y, 40–49 y, and 70+ y).

**Table 7:** Female and male NHANES body composition measures using a small dataset (*N* = 20) as if a single male participant was misgendered. Expected results compared to sex disparities in a small dataset were conducted using χ^2^.

	Sample Size	BMI (kg•m^−2^)	Subscapular Skinfold (mm)	Triceps Skinfold (mm)	Waist Girth (cm)	Body Mass (kg)
Female	7	26.9 (6.7)	16.7 (5.5)	21.9 (5.7)	85.2 (13.2)	64.7 (15.9)
Male	13	25.1 (3.9)	17.7 (7.1)	13.6 (6.7)	94.0 (12.6)	76.0 (13.1)
*p*-value		0.264	0.363	**0.006**	0.087	0.07
Cohen’s *d*		0.362 (small)	0.155 (small)	1.303 (large)	0.687 (medium)	0.799 (medium)
χ^2^ Result	χ^2^ (1, *N* = 10) = 13.52, ***p* < 0.001**, φ = 1.163 (large)					
18–29 Year						
Female	7	24.2 (4.4)	20.1 (7.0)	22.5 (7.1)	87.7 (13.6)	66.5 (13.7)
Male	13	27.6 (5.2)	20.4 (7.6)	16.3 (9.3)	94.0 (15.2)	84.2 (17.4)
*p*-value		0.073	0.458	0.056	0.179	**0.012**
Cohen’s *d*		0.685 (medium)	0.049 (small)	0.726 (medium)	0.430 (small)	1.087 (large)
χ^2^ Result	χ^2^ (1, *N* = 10) = 43.65, ***p* < 0.001**, φ = 2.100 (large)					
30–39 Year						
Female	7	20.9 (2.1)	14.4 (3.1)	17.3 (6.2)	76.2 (6.6)	55.7 (6.5)
Male	13	27.3 (4.6)	19.7 (7.2)	14.1 (8.1)	93.8 (12.5)	81.4 (15.8)
*p*-value		**0.001**	**0.04**	0.168	**< 0.001**	**< 0.001**
Cohen’s *d*		1.621 (large)	0.871 (large)	0.428 (small)	1.612 (large)	1.912 (large)
χ^2^ Result	χ^2^ (1, *N* = 10) = 26.44, ***p* < 0.001**, φ = 1.626 (large)					
40–49 Year						
Female	7	25.3 (4.5)	19.1 (9.1)	24.4 (9.0)	86.0 (12.2)	66.1 (12.9)
Male	13	28.3 (5.3)	22.4 (8.4)	17.6 (8.2)	99.0 (12.1)	84.4 (16.4)
*p*-value		0.105	0.226	0.063	**0.021**	**0.007**
Cohen’s *d*		0.584 (medium)	0.373 (small)	0.798 (medium)	1.067 (large)	1.199 (large)
χ^2^ Result	χ^2^ (1, *N* = 10) = 121.03, ***p* < 0.001**, φ = 3.479 (large)					
50–59 Year						
Female	7	25.6 (6.3)	22.4 (10.9)	26.3 (6.7)	88.3 (13.2)	65.4 (10.2)
Male	13	27.8 (4.4)	20.1 (7.2)	13.9 (8.1)	100.2 (9.5)	83.1 (12.4)
*p*-value		0.212	0.315	**0.001**	**0.032**	**0.002**
Cohen’s *d*		0.439 (small)	0.265 (small)	1.614 (large)	1.090 (large)	1.520 (large)
χ^2^ Result	χ^2^ (1, *N* = 10) = 0.515, *p* = 0.473, φ = 0.227 (small)					
60–69 Year						
Female	7	27.6 (4.7)	18.1 (10.6)	30.5 (7.1)	93.2 (9.7)	71.6 (9.4)
Male	13	29.5 (5.2)	23.0 (8.3)	18.2 (10.6)	105.6 (10.2)	88.1 (18.6)
*p*-value		0.21	0.074	**0.003**	**0.01**	**0.008**
Cohen’s *d*		0.376 (small)	0.645 (medium)	1.279 (large)	1.230 (large)	1.026 (large)
χ^2^ Result	χ^2^ (1, *N* = 10) = 0.56, *p* = 0.455, φ = 0.236 (small)					
70+ Year						
Female	7	26.7 (3.5)	16.7 (7.0)	21.5 (8.4)	96.5 (9.8)	68.2 (11.0)
Male	13	28.4 (5.1)	20.0 (9.1)	15.4 (7.8)	105.6 (10.5)	81.4 (14.0)
*p*-value		0.197	0.191	0.07	**0.038**	**0.017**
Cohen’s *d*		0.367 (small)	0.390 (small)	0.759 (medium)	0.883 (large)	1.010 (large)
χ^2^ Result	χ^2^ (1, *N* = 10) = 43.65, ***p* < 0.001**, φ = 2.100 (large)					

Values are means (standard deviations). Cohen’s *d* interpreted as small = 0.00–0.49, medium = 0.50–0.79, and large ≥ 0.80 [23]. Effect size j interpreted as trivial <0.1, small = 0.1–0.29, medium = 0.30–49, and large ≥ 0.5 [23]. Gray indicates a different decision or interpretation than initially made at baseline. NHANES: National Health and Nutrition Examination Survey; χ^2^: chi-squared; BMI: body mass index in kilograms(kg)•meter^−2^; mm: millimeters; cm: centimeters.

## Discussion

The purpose of this investigation was to utilize a large NHANES dataset to determine how statistical decisions might be altered when considering the effect of female recruiting disparities, small sample sizes, and the effect of misgendering on body composition measures. It was hypothesized that recruiting an imbalanced ratio of females to males, utilizing small sample sizes, and misgendering individuals would affect the ultimate interpretation of the data. Utilizing a small sample size comparable to what is employed in many sport and exercise science investigations [2, 12] affected both decisions (*t*-test results) and interpretations (effect size) to a greater extent than larger sample sizes. Furthermore, the potential effect of a sex recruiting disparity was amplified in a small sample size. Lastly, the effect of potential misgendering in sport and exercise science research is impactful regardless of the size of the sample recruited.

A number of authors have reported the consistent use of small sample sizes in sport and exercise science-related research [12, 13, 24]. To our knowledge, no investigations have systematically evaluated the intersection of sample size and sex recruiting disparity in sport and exercise science. Based on the results of the current analysis, the practice of recruiting primarily cisgender males does not appear to be impacted in large and robust sample sizes when considering body composition measures. However, when utilizing a small sample size and cisgender female ratio common in many sport and exercise science studies, the present data provide evidence that different interpretations occur for the overall sample, as well as almost every age category. While the consequences for sport and exercise science and related disciplines have yet to be determined, our data indicate different decisions may be made for the seemingly straightforward measures of body composition. Not confined to sport and exercise science, the clinical diagnosis and prevention of disease was derived almost exclusively from research on male cell lines, animals, and men [25]. Gender is also largely overlooked in technology and engineering [26]. A concerted effort to address this issue from a public health perspective has emerged, as the National Institutes of Health have required researchers to account for sex as a biological variable in funded preclinical research since 2016 [27, 28]. In sport and exercise science, the consequences of persistent underrepresentation are impactful, as cisgender females experience sport and exercise and associated recovery differently than males [1] and suffer from the dearth of appropriate scientific data to make informed decisions about modifying training-based variables [3]. The results of the current investigation raise the very real possibility of false positives in exercise-related disciplines (i.e., test result indicating the presence of a sex-based difference when there really is not one present), as well as the possibility of false negatives (i.e., test result indicating no sex-based difference when there really is one present) due to chronically small sample recruitment skewed against the inclusion of cisgender females.

To further determine the lack of inclusion in exercise-related research, we observed the effect of potential misgendering on the binary sex differences and interpretations associated with body composition metrics. We acknowledge the political hazard this issue has recently become [29] but do not feel ignoring individuals who identify as transgender or nonbinary is an appropriate course. The reporting of gender minorities appears to be the exception, as shown in the IJES self-study where, of over 151,000 participants, a single person was reported as transgender and three as other [2]. While likely not intentional, it is possible that many researchers are simply not allowing the possibility that a recruited individual in an exercise intervention could be transgender or nonbinary. This is problematic because the population presents with unique health disparities [30] that could become exacerbated by the wave of anti-transgender legislation [29]. Because these statutes imply that transgender people are not accepted where people live, work, and play [31], sport and exercise science researchers are urged to utilize inclusive methods for collecting gender data. Several resources exist in other fields that can be adapted for use in sport and exercise science investigations [32–36].

The findings of the current investigation provide evidence that misgendering affects decisions surrounding body composition metrics regardless of the sample size employed. While the current study employed conservative estimates for the percentage of transgender and nonbinary individuals [15, 16], these values are likely underestimated, given the stigma that gender minorities face [37]. It is understood that some investigators may be hesitant to include the data for a person they perceive to be in a misaligned category (i.e., a person assigned male at birth who identifies as a female into the female category, or a person assigned female at birth who identifies as a male into the male category). The effects of doing so will likely increase the variability of the dataset. The current findings show that, at least for measures associated with body composition, this variability will change ultimate decisions and interpretations of the data. However, variable data is inherent in sport and exercise-related disciplines [38–40]. Rather than treating this data as unwanted noise to be reduced, it is encouraged that sport and exercise scientists become comfortable with the variability in our data.

This investigation is not without limitations. The participants for each data set were collated from a random draw of the overall population which may not reflect samples utilized in sport and exercise science research, so the findings should be interpreted with caution. It is acknowledged that, particularly in smaller samples, the influence exerted by a single individual or groups of individuals could significantly change results and successive interpretation. Toward this end, a greater volume of simulations could be conducted to provide a probability to which the influence is expected to extend. Another limitation is that data from the current investigation was confined exclusively to body composition variables. Future studies of this nature could extend the scope to encompass a wider array of health-related variables.

In conclusion, these findings provide evidence that select practices in sport and exercise science testing and research should be revisited. First, the tendency of recruiting cisgender females in disparate numbers compared to cisgender males has consequences regarding ultimate decisions and interpretations, particularly in the small sample sizes that dominate the sport and exercise-related disciplines. A concerted effort should be made toward more equitable representation. Second, sport and exercise scientists should reevaluate the methods by which gender data are collected. Our findings provide evidence that misgendering has significant consequences on ultimate decisions and interpretations of data, regardless of the sample size employed. A relatively innocuous first step is providing more than traditional binary options when collecting the gender identity of participants. Inclusiveness is needed in helping all individuals feel valued and respected when participating in sport and exercise science-related research. Moreover, inclusive research will promote more equitable access to health-related data that is useful to all people, not only cisgender males.

## Supporting information

S1 Table Expanded tables for effects of sex disparities in the entire data, large (Large Ratio), and small (Small Ratio) datasets. (DOCX)
