# Supplementary material for: Implications for cisgender female underrepresentation, small sample sizes, and misgendering in sport and exercise science research
Source: PLoS One. 2023 Nov 30;18(11):e0291526. doi: 10.1371/journal.pone.0291526 (PMC10688738; doi:10.1371/journal.pone.0291526)
Supplement: S1 Table — (DOCX) [file pone.0291526.s001.docx]

**Table A.** Female and male NHANES body composition measures using the entire identified dataset as if a testing disparity was present for sex. Expected results compared to baseline were conducted using χ^2^.

|  | Sample Size | BMI (kg•m^−2^) | Subscapular Skinfold (mm) | Triceps Skinfold (mm) | Waist Girth (cm) | Body Mass (kg) |
| --- | --- | --- | --- | --- | --- | --- |
| Female | 948 | 25.9 (4.9) | 20.0 (7.7) | 22.8 (7.3) | 89.6 (12.5) | 67.1(13.2) |
| Male | 1,882 | 26.4 (4.3) | 18.6 (7.2) | 13.7 (6.1) | 95.6 (13.1) | 80.8 (15.2) |
| *p*-value |  | **0.05** | **< 0.001** | **< 0.001** | **< 0.001** | **< 0.001** |
| Cohen's *d* |  | 0.103 (small) | 0.180 (small) | 1.400 (large) | 0.466 (small) | 0.947 (large) |
| χ^2^ Result | χ^2^ (1, *N* = 10) = 0.52, *p* = 0.4729*,*  ϕ = 0.227 (small) | | | | |  |
| 18–29 Year |  |  |  |  |  |  |
| Female | 292 | 24.5 (4.5) | 18.8 (7.7) | 21.1 (7.4) | 85.5 (13.07) | 64.6 (12.6) |
| Male | 536 | 25.0 (4.7) | 16.0 (7.5) | 13.2 (7.0) | 87.6 (13.0) | 78.6 (16.3) |
| *p*-value |  | 0.064 | **< 0.001** | **< 0.001** | **< 0.001** | **< 0.001** |
| Cohen's *d* |  | 0.111 (small) | 0.368 (small) | 1.109 (large) | 0.166 (small) | 0.924 (large) |
| χ^2^ Result | χ^2^ (1, *N* = 10) = 0.56, *p* = 0.455*,* ϕ = 0.236 (small) | | | |  |  |
| 30–39 Year |  |  |  |  |  |  |
| Female | 151 | 25.8 (4.8) | 20.8 (7.8) | 23.5 (7.1) | 88.8 (12.1) | 68.7 (13.9) |
| Male | 265 | 26.6 (4.0) | 18.8 (6.6) | 13.4 (5.4) | 93.6 (11.0) | 81.4 (14.4) |
| *p*-value |  | **0.045** | **0.002** | **< 0.001** | **< 0.001** | **< 0.001** |
| Cohen's *d* |  | 0.173 (small) | 0.289 (small) | 1.657 (large) | 0.420 (small) | 0.895 (large) |
| χ^2^ Result | χ^2^ (1, *N* = 10) = 0.52, *p* = 0.4729*,*  ϕ = 0.227 (small) | | | | |  |
| 40–49 Year |  |  |  |  |  |  |
| Female | 112 | 26.9 (6.0) | 22.2 (8.2 | 24.7 (7.7) | 90.1 (13.1) | 69.6 (15.7) |
| Male | 269 | 27.1 (3.9) | 20.2 (7.0) | 13.8 (5.6) | 97.1 (10.7) | 83.8 (13.6) |
| *p*-value |  | 0.344 | **0.008** | **< 0.001** | **< 0.001** | **< 0.001** |
| Cohen's *d* |  | 0.045 (small) | 0.270 (small) | 1.718 (large) | 0.614 (medium) | 0.998 (large) |
| χ Result | χ^2^ (1, *N* = 10) = 0.56, *p* = 0.455*,* ϕ = 0.236 (small) | | | | |  |
| 50–59 Year |  |  |  |  |  |  |
| Female | 97 | 26.1 (4.7) | 22.1 (7.6) | 24.3 (6.8) | 90.1 (12.6) | 68.4 (11.8) |
| Male | 196 | 26.9 (4.1) | 20.1 (6.8) | 13.6 (5.7) | 99.5 (11.2) | 83.7 (14.4) |
| *p*-value |  | 0.069 | **0.012** | **< 0.001** | **< 0.001** | **< 0.001** |
| Cohen's *d* |  | 0.185 (small) | 0.281 (small) | 1.766 (large) | 0.796 (medium) | 1.124 (large) |
| χ^2^ Result | χ^2^ (1, *N* = 10) = 0.52, *p* = 0.4729*,*  ϕ = 0.227 (small) | | | | |  |
| 60–69 Year |  |  |  |  |  |  |
| Female | 128 | 27.3 (5.1) | 21.1 (7.1) | 24.5 (7.2) | 93.7 (11.4) | 69.7 (12.4) |
| Male | 231 | 27.7 (4.6) | 21.3 (6.8) | 14.5 (6.1) | 102.2 (12.2) | 84.2 (15.7) |
| *p*-value |  | 0.189 | 0.417 | **< 0.001** | **< 0.001** | **< 0.001** |
| Cohen's *d* |  | 0.097 (small) | 0.023 (small) | 1.536 (large) | 0.714 (medium) | 0.832 (large) |
| χ^2^ Result | χ^2^ (1, *N* = 10) = 0.52, *p* = 0.4729*,*  ϕ = 0.227 (small) | | | | |  |
| 70+ Year |  |  |  |  |  |  |
| Female | 168 | 26.6 (4.2) | 17.6 (7.0) | 22.0 (6.6) | 93.7 (10.1) | 65.4 (12.2) |
| Male | 385 | 26.6 (4.0) | 18.7 (6.5) | 14.1 (5.7) | 101.22 (11.3) | 78.1 (14.2) |
| *p*-value |  | 0.447 | **0.031** | **< 0.001** | **< 0.001** | **< 0.001** |
| Cohen's *d* |  | 0.012 (small) | 0.173 (small) | 1.310 (large) | 0.681 (medium) | 0.929 (large) |
| χ^2^ Result | χ^2^ (1, *N* = 10) = 0.52, *p* = 0.4729*,* ϕ = 0.227 (small) | | | |  |  |

Values are means (standard deviations). Cohen’s *d* interpreted as small = 0.00–0.49, medium = 0.50–0.79, and large ≥ 0.80 (23). Effect size ϕ interpreted as trivial < 0.1, small = 0.1–0.29, medium = 0.3­0–0.49, and large ≥ 0.5 (23). Gray indicates a different decision or interpretation than initially made at baseline in the overall sample. NHANES: National Health and Nutrition Examination Survey; χ^2^: chi-squared; BMI: body mass index in kilograms(kg)•meter^−2^; mm: millimeters; cm: centimeters.

**Table B.** Female and male NHANES body composition measures using a large dataset (*N* = 187) as if a testing disparity was present for sex. Expected results compared to baseline were conducted using χ^2^.

|  | Sample Size | BMI (kg•m^−2^) | Subscapular Skinfold (mm) | Triceps Skinfold (mm) | Waist Girth (cm) | Body Mass (kg) |  |
| --- | --- | --- | --- | --- | --- | --- | --- |
| Female | 62 | 26.2 (4.3) | 20.6 (7.9) | 22.9 (7.7) | 89.7 (11.2) | 67.7 (11.6) |  |
| Male | 125 | 25.9 (4.3) | 18.5 (7.3) | 13.5 (6.1) | 94.7 (12.9) | 78.7 (15.3) |  |
| *p*-value |  | 0.358 | **0.038** | **< 0.001** | **0.003** | **< 0.001** |  |
| Cohen's *d* |  | 0.057 (small) | 0.286 (small) | 1.411 (large) | 0.408 (small) | 0.774 (medium) |  |
| χ^2^ Result | χ^2^ (1, *N* = 10) = 4.89, ***p* = 0.027***,* ϕ = 0.700 (large) | | | |  |  |  |
| 18–29 Year |  |  |  |  |  |  |  |
| Female | 62 | 24.4 (5.0) | 18.6 (9.1) | 21.3 (8.6) | 84.1 (13.5) | 65.2 (14.3) |  |
| Male | 125 | 25.5 (5.2) | 16.5 (8.1) | 13.9 (7.6) | 88.6 (14.0) | 78.6 (17.8) |  |
| *p*-value |  | 0.087 | 0.065 | **< 0.001** | **0.017** | **< 0.001** |  |
| Cohen's *d* |  | 0.209 (small) | 0.246 (small) | 0.927 (large) | 0.327 (small) | 0.804 (large) |  |
| χ^2^ Result | χ^2^ (1, *N* = 10) = 0.56, *p* = 0.455*,* ϕ = 0.236 (small) | | | |  |  |  |
| 30–39 Year |  |  |  |  |  |  |  |
| Female | 62 | 25.1 (4.3) | 20.2 (7.4) | 22.2 (6.5) | 88.3 (11.5) | 66.5 (12.0) |  |
| Male | 125 | 25.9 (3.8) | 18.1 (6.6) | 12.7 (5.0) | 92.0 (11.0) | 78.9 (13.5) |  |
| *p*-value |  | 0.098 | **0.029** | **< 0.001** | **0.019** | **< 0.001** |  |
| Cohen's *d* |  | 0.210 (small) | 0.310 (small) | 1.704 (large) | 0.331 (small) | 0.955 (large) |  |
| χ^2^ Result | χ (1, *N* = 10) = 0.56, *p* = 0.455, ϕ = 0.236 (small) | | | |  |  |  |
| 40–49 Year |  |  |  |  |  |  |  |
| Female | 62 | 26.8 (4.8) | 21.5 (7.9) | 25.5 (7.4) | 90.2 (12.1) | 71.4 (14.6) |  |
| Male | 125 | 27.8 (4.2) | 21.0 (7.0) | 14.3 (6.0) | 98.8 (11.2) | 85.5 (15.0) |  |
| *p*-value |  | 0.086 | 0.327 | **< 0.001** | **< 0.001** | **< 0.001** |  |
| Cohen's *d* |  | 0.225 (small) | 0.073 (small) | 1.723 (large) | 0.744 (medium) | 0.946 (large) |  |
| χ^2^ Result | χ^2^ (1, *N* = 10) = 0.56, *p* = 0.455*,* ϕ = 0.236 (small) | | | |  |  |  |
| 50–59 Year |  |  |  |  |  |  |  |
| Female | 62 | 26.5 (5.5) | 22.5 (8.2) | 24.6 (7.5) | 91.6 (12.8) | 69.6 (13.8) |  |
| Male | 125 | 26.7 (4.0) | 19.7 (6.6) | 13.2 (5.8) | 98.8 (11.2) | 83.0 (14.1) |  |
| *p*-value |  | 0.354 | **0.007** | **< 0.001** | **< 0.001** | **< 0.001** |  |
| Cohen's *d* |  | 0.058 (small) | 0.390 (small) | 1.777 (large) | 0.615 (medium) | 0.957 (large) |  |
| χ^2^ Result | χ^2^ (1, *N* = 10) = 0.52, *p* = 0.4729*,* ϕ = 0.227 (small) | | | |  |  |  |
| 60–69 Year |  |  |  |  |  |  |  |
| Female | 62 | 28.3 (4.7) | 22.8 (8.1) | 25.5 (7.1) | 96.8 (10.4) | 73.3 (13.2) |  |
| Male | 125 | 28.0 (4.6) | 21.2 (6.3) | 14.6 (5.7) | 102.7 (12.0) | 85.1 (15.9) |  |
| *p*-value |  | 0.343 | 0.067 | **< 0.001** | **< 0.001** | **< 0.001** |  |
| Cohen's *d* |  | 0.063 (small) | 0.233 (small) | 1.763 (large) | 0.520 (medium) | 0.785 (medium) |  |
| χ^2^ Result | χ^2^ (1, *N* = 10) = 0.56, *p* = 0.455*,* ϕ = 0.236 (small) | | | |  |  |  |
| 70+ Year |  |  |  |  |  |  |  |
| Female | 62 | 26.5 (4.1) | 18.2 (7.0) | 22.0 (7.1) | 95.0 (10.2) | 65.0 (11.4) |  |
| Male | 125 | 26.1 (4.1) | 17.8 (6.1) | 13.6 (5.8) | 99.6 (11.0) | 75.7 (13.2) |  |
| *p*-value |  | 0.283 | 0.341 | **< 0.001** | **0.003** | **< 0.001** |  |
| Cohen's *d* |  | 0.090 (small) | 0.067 (small) | 1.351 (large) | 0.429 (small) | 0.844 (large) |  |
| χ^2^ Result | χ^2^ (1, *N* = 10) = 0.56, *p* = 0.455*,* ϕ = 0.236 (small) | | | |  |  |  |

Values are means (standard deviations). Cohen’s *d* interpreted as small = 0.00–0.49, medium = 0.50–0.79, and large ≥ 0.80 (23). Effect size ϕ interpreted as trivial < 0.1, small = 0.1–0.29, medium = 0.3­0–0.49, and large ≥ 0.5 (23). Gray indicates a different decision or interpretation than initially made at baseline. NHANES: National Health and Nutrition Examination Survey; χ^2^: chi-squared; BMI: body mass index in kilograms(kg)•meter^−2^; mm: millimeters; cm: centimeters.

**Table C.** Female and male NHANES body composition measures using a small dataset (*N* = 20) as if a testing disparity was present for sex. Expected results compared to baseline were conducted using χ^2^.

|  | Sample Size | BMI (kg•m^−2^) | Subscapular Skinfold (mm) | Triceps Skinfold (mm) | Waist Girth (cm) | Body Mass (kg) |
| --- | --- | --- | --- | --- | --- | --- |
| Female | 7 | 25.4 (4.3) | 19.1 (7.4) | 20.9 (8.4) | 90.2 (11.7) | 67.1 (13.2) |
| Male | 13 | 25.8 (5.2) | 16.5 (8.0) | 13.6 (7.4) | 93.0 (15.6) | 80.6 (18.4) |
| *p*–value |  | 0.438 | 0.242 | **0.04** | 0.329 | **0.038** |
| Cohen's *d* |  | 0.071 (small) | 0.329 (small) | 0.939 (large) | 0.194 (small) | 0.801 (large) |
| χ^2^ Result | χ^2^ (1, *N* = 10) = 13.52, ***p* < 0.001***,* ϕ = 1.163 (large) | | | |  |  |
| 18–29 Year |  |  |  |  |  |  |
| Female | 7 | 22.1 (2.6) | 15.9 (4.0) | 16.8 (3.3) | 79.4 (10.3) | 54.7 (6.6) |
| Male | 13 | 25.9 (4.7) | 17.0 (5.9) | 14.7 (7.3) | 91.5 (16.5) | 79.0 (18.1) |
| *p*-value |  | **0.017** | 0.331 | 0.236 | **0.03** | **< 0.001** |
| Cohen's *d* |  | 0.910 (large) | 0.209 (small) | 0.334 (small) | 0.821 (large) | 1.587 (large) |
| χ^2^ Result | χ^2^ (1, *N* = 10) = 43.65, ***p* < 0.001***,* ϕ = 2.100 (large) | | | |  |  |
| 30–39 Year |  |  |  |  |  |  |
| Female | 7 | 25.4 (3.9) | 22.6 (7.3) | 24.1 (7.1) | 86.1 (9.3) | 67.1 (10.6) |
| Male | 13 | 28.8 (4.0) | 19.0 (6.4) | 14.3 (4.8) | 98.3 (11.7) | 89.4 (13.1) |
| *p*-value |  | **0.045** | 0.152 | **0.005** | **0.011** | **< 0.001** |
| Cohen's *d* |  | 0.851 (large) | 0.529 (medium) | 1.732 (large) | 1.113 (large) | 1.814 (large) |
| χ^2^ Result | χ^2^ (1, *N* = 10) = 43.65, ***p* < 0.001** ϕ = 2.100 (large) | | | |  |  |
| 40–49 Year |  |  |  |  |  |  |
| Female | 7 | 28.7 (4.9) | 22.6 (10.5) | 26.6 (9.1) | 94.3 (11.8) | 77.4 (16.4) |
| Male | 13 | 26.8 (4.4) | 17.1 (6.1) | 11.8 (6.7) | 96.3 (12.5) | 84.2 (13.6) |
| *p*-value |  | 0.207 | 0.116 | **0.002** | 0.367 | 0.178 |
| Cohen's *d* |  | 0.412 (small) | 0.710 (medium) | 1.967 (large) | 0.159 (small) | 0.50 (medium) |
| χ^2^ Result | χ^2^ (1, *N* = 10) = 65.15, ***p* < 0.001***,*ϕ = 2.552 (large) | | | |  |  |
| 50–59 Year |  |  |  |  |  |  |
| Female | 7 | 25.2 (3.5) | 19.4 (6.4) | 22.6 (8.0) | 85.5 (9.4) | 64.2 (8.2) |
| Male | 13 | 27.2 (4.1) | 20.2 (6.3) | 13.2 (4.1) | 98.8 (9.2) | 82.9 (13.8) |
| *p*-value |  | 0.142 | 0.403 | **0.001** | **0.005** | **0.001** |
| Cohen's *d* |  | 0.497 (small) | 0.119 (small) | 1.638 (large) | 1.441 (large) | 1.675 (large) |
| χ^2^ Result | χ^2^ (1, *N* = 10) = 4.89, ***p* = 0.027***,* ϕ = 0.700 (large) | | | |  |  |
| 60–69 Year |  |  |  |  |  |  |
| Female | 7 | 26.5 (3.3) | 19.4 (6.2) | 28.6 (9.3) | 89.2 (11.6) | 65.5 (6.0) |
| Male | 13 | 28.2 (4.4) | 20.5 (6.9) | 15.4 (4.9) | 103.6 (7.9) | 85.1 (13.1) |
| *p*-value |  | 0.168 | 0.364 | **0.004** | **0.008** | **< 0.001** |
| Cohen's *d* |  | 0.423 (small) | 0.162 (small) | 1.977 (large) | 1.542 (large) | 1.753 (large) |
| χ^2^ Result | χ^2^ (1, *N* = 10) = 0.56, *p* = 0.455*,* ϕ = 0.236 (small) | | | |  |  |
| 70+ Year |  |  |  |  |  |  |
| Female | 7 | 26.2 (4.3) | 18.1 (6.9) | 23.2 (8.3) | 95.7 (8.2) | 63.0 (11.6) |
| Male | 13 | 25.3 (3.6) | 17.9 (9.4) | 13.6 (5.1) | 97.0 (10.0) | 72.3 (10.6) |
| *p*-value |  | 0.31 | 0.482 | **0.011** | 0.376 | 0.053 |
| Cohen's *d* |  | 0.252 (small) | 0.020 (small) | 1.508 (large) | 0.142 (small) | 0.845 (large) |
| χ^2^ Result | χ^2^ (1, *N* = 10) = 26.44, ***p* < 0.001***,* ϕ = 1.626 (large) | | | |  |  |

Values are means (standard deviations). Cohen’s *d* interpreted as small = 0.00–0.49, medium = 0.50–0.79, and large ≥ 0.80 (23). Effect size ϕ interpreted as trivial < 0.1, small = 0.1–0.29, medium = 0.3­0–0.49, and large ≥ 0.5 (23). Gray indicates a different decision or interpretation than initially made at baseline. NHANES: National Health and Nutrition Examination Survey; χ^2^: chi-squared; BMI: body mass index in kilograms(kg)•meter^−2^; mm: millimeters; cm: centimeters.
